# Identifying the Role of Oxidative Stress-Related Genes as Prognostic Biomarkers and Predicting the Response of Immunotherapy and Chemotherapy in Ovarian Cancer

**DOI:** 10.1155/2022/6575534

**Published:** 2022-12-12

**Authors:** Qingyang Liu, Xiaocheng Yang, Yuhan Yin, Hao Zhang, Fanxing Yin, Panpan Guo, Xiaoxu Zhang, Chenxi Sun, Shining Li, Yanshuo Han, Zhuo Yang

**Affiliations:** ^1^School of Life and Pharmaceutical Sciences, Dalian University of Technology, Panjin, China; ^2^Department of Gynecology, Cancer Hospital of Dalian University of Technology (Liaoning Cancer Hospital & Institute), Shenyang, China

## Abstract

**Background:**

Ovarian cancer (OC) is one of the most frequently seen and fatal gynecological malignancies, and oxidative stress (OS) plays a critical role in the development and chemoresistance of OC.

**Materials and Methods:**

OS-related genes (OSRGs) were obtained from the Molecular Signatures Database. Besides, gene expression profiles and clinical information from The Cancer Genome Atlas (TCGA) were selected to identify the prognostic OSRGs. Moreover, univariate Cox regression, LASSO, and multivariate Cox regression analyses were conducted sequentially to establish a prognostic signature, which was later validated in three independent Gene Expression Omnibus (GEO) datasets. Next, gene set enrichment analysis (GSEA) and tumor mutation burden (TMB) analysis were performed. Afterwards, immune checkpoint genes (ICGs) and the tumor immune dysfunction and exclusion (TIDE) algorithm, together with IMvigor210 and GSE78220 cohorts, were applied to comprehensively explore the role of OSRG signature in immunotherapy. Further, the CellMiner and Genomics of Drug Sensitivity in Cancer (GDSC) databases were also applied in investigating the significance of OSRG signature in chemotherapy.

**Results:**

Altogether, 34 prognostic OSRGs were identified, among which 14 were chosen to establish the most valuable prognostic signature. The Kaplan-Meier (KM) analysis suggested that patients with lower OS-related risk score had better prognosis. The area under the curve (AUC) values were 0.71, 0.76, and 0.85 in 3, 5, and 7 years separately, and the stability of this prognostic signature was confirmed in three GEO datasets. As revealed by GSEA and TMB analysis results, OC patients in low-risk group might have better immunotherapeutic response, which was consistent with ICG expression and TIDE analyses. Moreover, both IMvigor210 and GSE78220 cohorts demonstrated that patients with lower OS-related risk score were more likely to benefit from anti-PD-1/L1 immunotherapy. In addition, the association between prognostic signature and drug sensitivity was explored.

**Conclusion:**

According to our results in this work, OSRG signature can act as a powerful prognostic predictor for OC, which contributes to generating more individualized therapeutic strategies for OC patients.

## 1. Introduction

Ovarian cancer (OC) is a fatal female malignancy, and epithelial ovarian cancer (EOC) is its most essential pathological subtype, which ranks the fourth place among the causes of cancer death in women in developed countries [1, 2]. According to the Global Cancer Statistics 2020, the incidence and mortality rates of OC reported worldwide rank the eighth place among female cancers [3]. OC has been classified into stages I-IV by the International Federation of Gynecology and Obstetrics (FIGO) classification system [4]. Although some screening methods such as transvaginal ultrasonography and biomarkers like human epididymis protein 4 (HE4) and serum cancer antigen 125 (CA125) can screen early OC patients to a certain extent, only less than half of the diagnosed patients can survive more than five years or longer. This is usually due to the lack of detectable preinvasive phase and reliable biomarkers in OC; as a result, over 75% of patients are diagnosed at the advanced stage (FIGO stages III-IV) [1, 5–7]. With the development of surgery, platinum-based adjuvant therapy, antiangiogenic treatment, molecularly targeted treatment, and hormone replacement therapy, the therapeutic effect of patients has improved greatly. However, the survival rate cannot significantly increase because of the factors such as disease recurrence, secondary adverse reactions, and drug sensitivity [2, 8–11]. In addition, the prognosis of OC is strongly linked to the disease diagnosis stage; to be specific, the 5-year survival rates of patients diagnosed at stages I-IV are 93.3%, 67.7%, 26.9%, and 13.4%, respectively [12]. Given the difficulty in early diagnosis and the heterogeneous prognosis of OC, it is of great importance to investigate the carcinogenic mechanisms, identify better prognostic indicators, and develop individualized treatment for OC patients.

In healthy organisms, reactive oxygen species (ROS) and reactive nitrogen species (RNS), the regular by-products of biological metabolism, are concerned with the transduction of various signaling pathways and the regulation of growth factors, transcription, and hormones. Generally, the production of ROS and RNS is balanced with multiple antioxidant defenses [13, 14]. However, when the organism is stimulated by endogenous factors (such as cellular aerobic metabolism, inflammation, and enzymatic reaction) or exogenous factors (like X radiation, ultraviolet radiation, and environmental temperature changes), the excessive amounts of ROS and RNS overpower the organism's antioxidant defense system, conducing to oxidative damage in cells and tissues, a process called oxidative stress (OS) [15, 16]. To avoid excessive ROS and RNS production, some compounds including flavonoids, catalase (CAT), glutathione (GSH), thioredoxin (TXN), and reduced nicotinamide adenine dinucleotide phosphate (NADPH) play important roles in countering OS [17–19]. The concentrations of prooxidants have dual effects on the occurrence and progression of cancers [20]. Specifically, excessive ROS and RNS amounts promote the occurrence, progression, metastasis, and chemoresistance of cancers [20, 21], whereas the higher ROS and RNS levels have cytotoxic effects, which can suppress tumor development through inducing apoptosis and other pathways [22]. Previous studies have indicated that OS is associated with numerous diseases, including diabetes mellitus (DM) [23], gliomas [24], leukemia [25], and OC [26]. The majority of OC is caused by the interaction between environmental factors and genetic factors [1], so ROS and RNS levels may be the significant factors.

OS has multiple and complex effects on OC. According to relevant evidence, OS levels are normally much higher in OC patients due to the imbalance of antioxidant mechanisms [27–29]. Moreover, oxidative damage and mutation of deoxyribonucleic acid (DNA) bases caused by OS are considered as the pivotal factors for the progression of multiple cancers, like breast cancer (BC) and hepatocellular carcinoma (HCC) [30]. In particular, some study shows that hydroxyl radical generated by Fenton reaction can be the inducing molecule of DNA double-strand breaks (DSB) in fallopian tubal epithelium, which can stimulate the progression of OC [31]. More importantly, various signaling pathways modified by redox, including Wnt/*β*-catenin signaling pathway [32], AKT/mTOR signaling pathway [33], Nrf2/PGC1*α* signaling pathway [34], and Notch signaling pathway [35], have been proved to play vital roles in the pathogenesis of OC. For instance, the nucleoredoxin oxidized by ROS can activate Wnt/*β*-catenin signaling pathway [36], while the activated Wnt/*β*-catenin signaling pathway has been confirmed to be related to the enhanced platinum resistance in OC [37]. OS is also involved in OC development by affecting immune cells and metabolites in tumor microenvironment (TME) [38, 39]. Compared with healthy women, neutrophils have intensified functional activities in advanced OC patients, and significantly increased amounts of ROS are generated due to stimuli [40]. As suggested in the previous studies, OS has a certain impact on the therapeutic effect in OC patients. To be specific, OS can affect chemoresistance through specific point mutations of key redox enzymes [41]. Besides, ROS is one of an important second messengers of immune cells, which provides an opportunity for applying antioxidants in immunomodulatory therapy [42]. The abovementioned evidence strongly suggests that OS level affects the prognosis and treatment of OC patients. Therefore, identifying the latent value of OS-related genes (OSRGs) is momentous for predicting clinical outcomes and providing novel therapeutic strategies for OC patients. However, to our knowledge, no previous studies have focused on screening OSRGs as the biomarkers and constructing the prognostic model of OC.

In this study, data from The Cancer Genome Atlas (TCGA), Molecular Signatures Database (MSigDB), and Gene Expression Omnibus (GEO) databases were integrated to construct and validate an original prognostic signature based on OSRGs. On this basis, the differences in TME, immune cell infiltration, signaling pathways, and tumor mutation burden (TMB) between the high- and low-risk patients were comprehensively analyzed, which provided various novel immunotherapy and chemotherapy strategies for OC.

## 2. Materials and Methods

### 2.1. Data Collection and Processing

**Figure 1:**
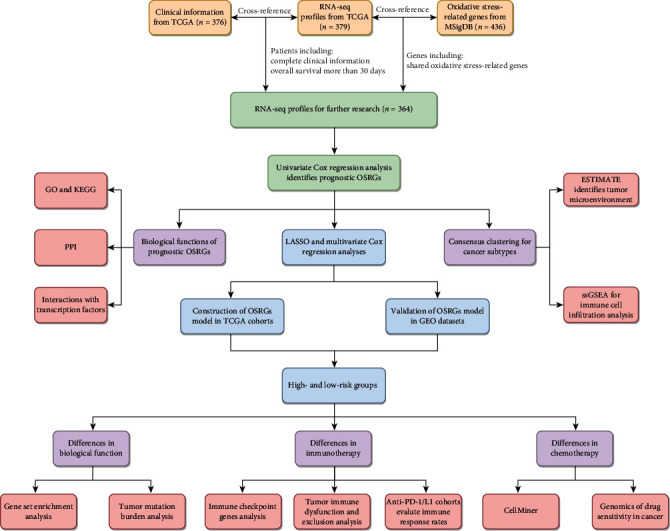
The flow chart of this study.

In this study, the RNA-seq profiles (FPKM) of OC patients and relevant clinical information were acquired from TCGA data portal (https://portal.gdc.cancer.gov). Besides, GSE14764, GSE63885, and GSE23554 datasets from the GEO database (http://www.ncbi.nlm.nih.gov/geo) were selected as the external validation datasets, whereas GSE78220 and IMvigor210 cohorts were selected to testify the effect of OSRG signature on immunotherapy. The “GOBP_RESPONSE_TO_OXIDATIVE_STRESS” gene set containing 436 OSRGs was obtained from MSigDB (https://www.gsea-msigdb.org/gsea/msigdb). For further analysis, TCGA-derived OC patients with complete clinical information and overall survival >30 days were retained. Finally, the integrated RNA-seq profiles involved 364 OC patients and 423 OSRGs. The detailed design process of this study is shown in Figure 1.

### 2.2. Differentially Expressed Gene (DEG) Identification

R package “limma” (version 3.47.16) was employed to discover DEGs. Adjusted *P* < 0.05 and |log2FoldChange(*FC*)| > 0.5 were set as the cutoff values to select significant DEGs. Moreover, DEGs were visualized by R package “pheatmap” (version 1.0.12) and “ggplot2” (version 3.3.3).

### 2.3. Functional Annotation

To investigate the potential signaling pathways and biological functions enriched by the target gene sets, R package “clusterProfiler” (version 3.99.2) was utilized for gene set enrichment analysis (GSEA) and Gene Ontology (GO) functional annotation and Kyoto Encyclopedia of Genes and Genomes (KEGG) pathway enrichment analyses. The enrichment results were visualized by R package “enrichplot” (version 1.11.3) and “ggplot2” (version 3.3.3), with adjusted *P* < 0.05 being the threshold of significance.

### 2.4. Construction of a Protein-Protein Interaction (PPI) Network

The Search Tool for the Retrieval of Interacting Genes/Proteins (STRING) database (http://www.string-db.org/) was applied to establish the PPI network based on prognostic OSRGs, and the network was drawn with Cytoscape.

### 2.5. Establishment and Verification of the Prognostic Signature

OSRGs with potential prognostic value were identified by univariate Cox regression analysis, with *P* < 0.05 being the threshold to select prognostic OSRGs. The Least Absolute Shrinkage and Selection Operator (LASSO), a kind of regression algorithm preserving valuable variables and avoiding overfitting [43], was utilized to shrink the number of OSRGs and to select the most valuable prognostic signature from all those identified OSRGs. Thereafter, the multivariate Cox regression algorithm was utilized to calculate individual-level risk scores. For each OC patient, the risk score represented the sum of the product of the expression levels of prognostic signature genes and corresponding coefficients obtained by multivariate Cox regression analysis:

(1) $$\begin{array}{c} \text{Risk score}=\overset{n}{\underset{i=1}{\sum }}\left(\beta_{i}\times {\text{Exp}}_{i}\right), \end{array}$$

where *β*_*i*_ represents the coefficient of signature gene *i*, Exp_*i*_ indicates the expression level of signature gene *i*, and *n* suggests the total number of signature genes. R package “survival” (version 3.2.11) and R package “glmnet” (version 4.1.3) were employed for univariate and multivariate Cox regression analyses and LASSO regression analysis, respectively.

Thereafter, the accuracy and robustness of this prognostic signature were evaluated. Firstly, R package “survminer” (version 0.4.9) was adopted to draw the Kaplan-Meier (KM) survival curves, which revealed the difference in patient survival between different groups. Moreover, R package “timeROC” (version 0.4) was applied in drawing the time-dependent receiver operating characteristic (t-ROC) curves. Besides, GSE14764, GSE23554, and GSE63885 datasets were chosen as the external datasets to further validate this prognostic signature.

### 2.6. Cancer Subtype

Consensus clustering (CC), a class discovery algorithm specific to gene expression data, allows to efficiently discover biologically meaningful clusters [44]. In this study, R package “CancerSubtypes” (version 1.17.1) was employed to apply the CC algorithm in prognostic OSRGs, so as to identify distinct OS-related patterns. Afterwards, the optimal cluster number was determined by consensus heatmap, cumulative distribution function (CDF) curve, and silhouette width.

### 2.7. Calculation of Immune, Stromal, and Estimate Scores

The ESTIMATE algorithm can utilize gene expression signatures to deduce the proportions of immune and stromal components in TME [45]. In this work, immune scores, stromal scores, and estimate scores were calculated by R package “estimate” (version 1.0.13), respectively.

### 2.8. Tumor Mutation Burden Analysis

TMB is a useful biomarker for predicting the immunotherapeutic response. Generally speaking, highly mutated tumors are more likely to contain neoantigens and respond to immune checkpoint inhibitors (ICIs) [46]. The present study adopted R package “maftools” (version 2.8.5) to calculate and visualize TMB in TCGA-derived OC samples.

### 2.9. Immune Cell Infiltration and Immune Checkpoint Gene Analyses

By using R package “GSVA” (version 1.40.0), the immune cell infiltration levels in OC patients were evaluated by single-sample gene set enrichment analysis (ssGSEA). The characteristic gene sets containing 28 immune cell subsets were acquired from the research by Charoentong et al. [47]. Besides, the relations between the immune cell infiltration levels and the expression levels of prognostic OSRGs were also explored.

Immune checkpoint genes (ICGs) play essential parts in immunotherapeutic effect. In this regard, 16 previously reported ICGs, including *B7-H3*, *CD27*, *CD270*, *CD40*, *CD58*, *CD70*, *CD86*, *CTLA4*, *ICOS*, *IDO1*, *LAG3*, *PD-1*, *PD-L1*, *PD-L2*, *TIGIT*, and *TIM-3* [48], were selected to analyze the differences in their expression levels between patients in high- and low-risk groups, which shed new lights on the immunotherapy for OC. In addition, the Spearman correlation analysis was conducted to explore the relations between these ICGs and OS-related risk score.

### 2.10. Prediction of Immunotherapeutic Response

The tumor immune dysfunction and exclusion (TIDE) algorithm (http://tide.dfci.harvard.edu) was utilized to predict the response to ICIs in each OC patient [49]. In terms of parameter selection, cancer type was selected as melanoma, whereas the previous immunotherapy as Yes. Moreover, IMvigor210 and GSE78220 cohorts were obtained to explore the ability of the as-constructed OSRG-based prognostic signature in predicting clinical response to immunotherapy.

### 2.11. Drug Sensitivity Analysis

The CellMiner database (https://discover.nci.nih.gov/cellminer/home.do) provides us with various drugs approved by clinically tested and by the Food and Drug Administration (FDA) together with drug activity levels [50]. High-grade serous OC has been widely reported to benefit from platinum-based drugs [51]. Consequently, this study focused on three platinum-based drugs (cisplatin, carboplatin, and oxaliplatin) obtained from the CellMiner database, so as to unveil the relations between the 50% growth inhibitory level (GI_50_/IC_50_) of platinum-based drugs and the expression levels of signature genes. Moreover, the Genomics of Drug Sensitivity in Cancer (GDSC) database (https://www.cancerrxgene.org/), one of the largest public databases for cancer drugs, has combined large-scale drug sensitivity datasets and genomic datasets [52]. Therefore, R package “oncoPredict” (version 0.2) was adopted to predict the difference in IC_50_ values of various drugs between the high- and low-risk patients.

### 2.12. Statistical Analysis

All statistical analyses were performed with R (Version 4.1.0). The Spearman correlation analysis was conducted to explore the relation between the two continuous variables, while log-rank test was applied to determine the significance of survival curves. Moreover, the Wilcoxon test was utilized to compare the paired independent samples, and the Kruskal-Wallis test was used to compare three or more independent samples.

## 3. Results

### 3.1. Identification and Biological Functions of Prognostic OSRGs

**Figure 2:**
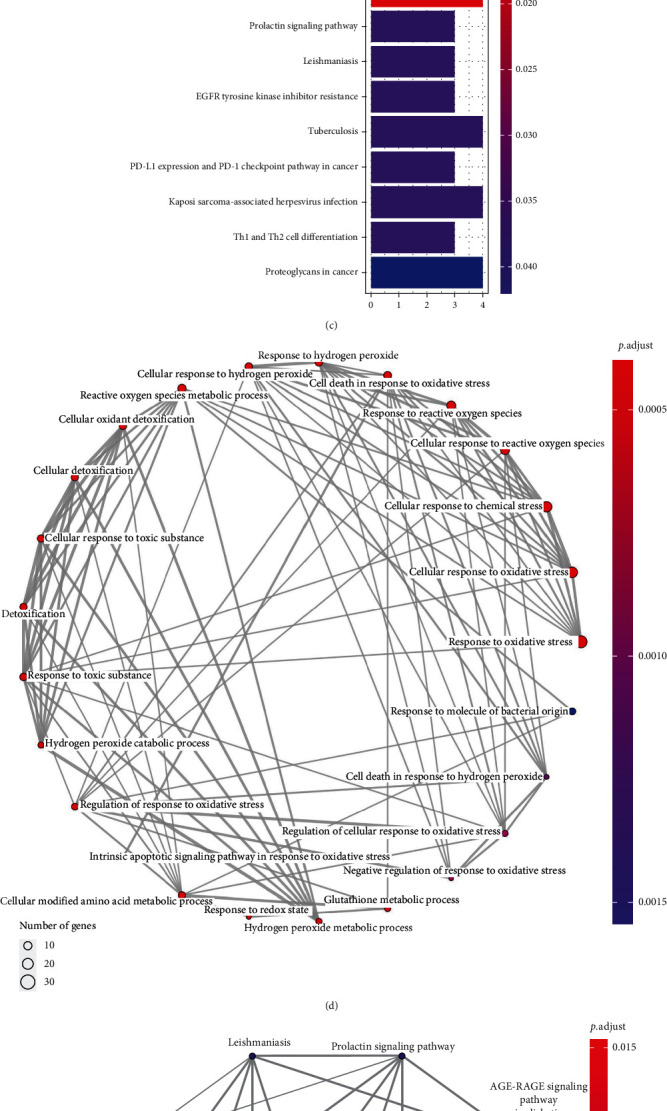
Overview for biological functions of prognostic OSRGs. (a) Forest map showing the prognostic information of 34 OSRGs. Hazard ratios (HR) < 1 represent favorable prognosis, while HR > 1 indicate poor prognosis. (b) Histogram presenting GO functional annotation on prognostic OSRGs. (c) Histogram showing KEGG enrichment analysis on prognostic OSRGs. (d) Interactions of the top 25 GO terms. (e) Interactions of the top 10 KEGG pathways. (f) The PPI network constructed based on prognostic OSRGs. (g) Interactions between prognostic OSRGs (green) and transcription factors (orange).

Based on the integrated gene expression profiles involving 364 TCGA-derived OC samples and 423 OSRGs, 384 OSRGs were retained after removing genes with low expression levels. Subsequently, univariate Cox regression analysis was conducted to identify prognostic OSRGs, and finally, 34 OSRGs were selected for subsequent analysis (*P* < 0.05) (Figure 2(a), Supplemental Table 1).

To investigate the latent biological functions of these OSRGs, GO and KEGG enrichment analyses were carried out at first. As revealed by GO functional annotation, these OSRGs were mainly enriched in cellular response to oxidative stress, chemical stress, reactive oxygen species, hydrogen peroxide, and cell death in response to oxidative stress in terms of biological process (BP) category; while with regard to molecular function (MF) category, these prognostic OSRGs were enriched in antioxidant, peroxidase, oxidoreductase, receptor antagonist, protein tyrosine kinase, hydrolase, and binding of histone acetyltransferase and heme (Figure 2(b)). Based on KEGG pathway enrichment analysis, 34 OSRGs were mainly enriched into pathways like nicotinate and nicotinamide metabolism, EGFR tyrosine kinase inhibitor resistance, Th1 and Th2 cell differentiation, prolactin signaling pathway, PD-L1 expression and PD-1 checkpoint pathway in cancer, and proteoglycans in cancer (Figure 2(c)). The results of GO and KEGG analyses are, respectively, displayed in Figures 2(d) and 2(e). To sum up, these results demonstrated that prognostic OSRGs were closely associated with ROS generation, various metabolic processes regulating the occurrence and progression of tumors, drug resistance, and immune response.

Afterwards, the PPI network incorporating 34 prognostic OSRGs was constructed based on the STRING database, which was further visualized by Cytoscape (Figure 2(f)). As a result, there were 34 nodes and 91 edges in this network, and some key proteins such as *STAT1*, *DUOX1*, *PRDX6*, *JAK2*, *CD38*, *FOXO1*, *HGF*, *SRXN1*, and *SIRT2* might play key roles in the oncogenesis of OC.

According to the previous studies, some transcription factors (TFs) can act as the regulators of cellular defense mechanisms to prevent OS [53]. Therefore, TFs related to prognostic OSRGs were predicted through the TRRUST database. According to the results, there were some interactions between the eight prognostic OSRGs and eight TFs (Figure 2(g)).

### 3.2. Consensus Clustering for Identifying Different Cancer Subtypes

**Figure 3:**
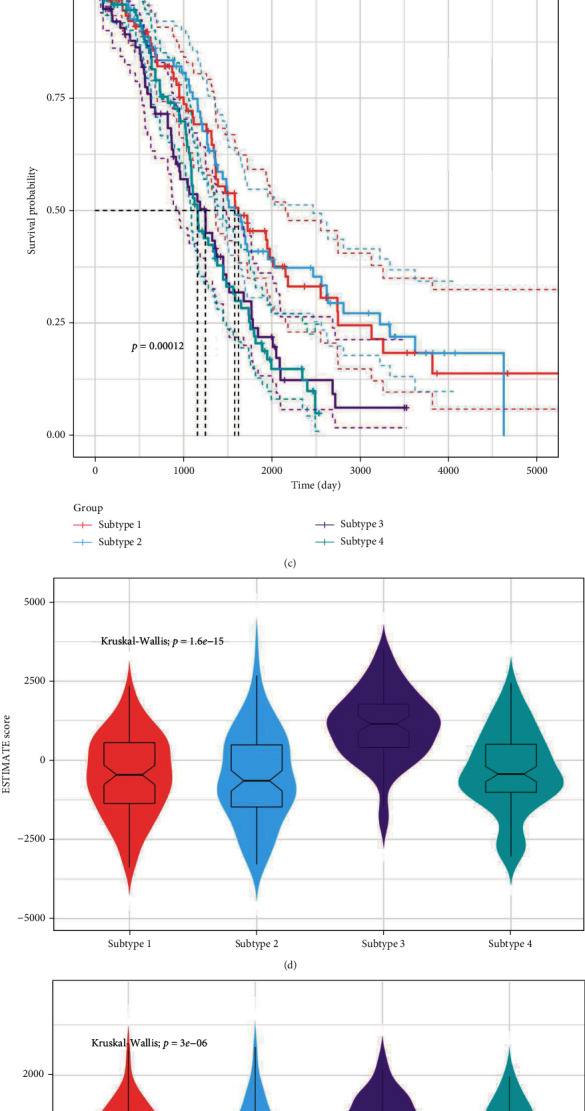
Tumor microenvironment in different OC subtypes. (a) Consensus heatmap showing four subtypes. (b) Silhouette width plot of four subtypes. (c) Survival curve of four subtypes. (d–f) The tumor purity and proportions of immune and stromal constituents between different subtypes evaluated by the ESTIMATE algorithm. (g) The correlation matrix between the three ESTIMATE scores and 34 prognostic OSRGs. ^∗^: *P* ≤ 0.05; ^∗∗^: *P* ≤ 0.01; ^∗∗∗^: *P* ≤ 0.001.

Taking 34 prognostic OSRGs as the characteristic genes, cancer subtypes were identified by the consensus clustering algorithm with R package “CancerSubtypes.” The number of candidate clusters was set as 2-10, while the optimal number of clusters was decided by overall consideration of consensus heatmap, consensus cumulative distribution function (CDF) curves, and silhouette width. As a result, compared with other numbers of clusters, consensus heatmap and CDF curve were more effective and the average silhouette width also presented sufficient robustness when OC samples were divided into four clusters (Figures 3(a) and 3(b) and Supplemental Figure 1a-l). Besides, the KM survival curves unveiled that patients in subtype1 and subtype2 had noticeably greater survival ability than those in subtype3 and subtype4 (Figure 3(c)). Therefore, four clusters were selected as the final cancer subtypes, namely, subtype1 (*n* = 98 patients), subtype2 (*n* = 91), subtype3 (*n* = 78), and subtype4 (*n* = 97).

### 3.3. Differences in Tumor Immune Microenvironment (TIME) among the Four Subtypes

Previous studies have suggested that OS is the key factor for immune cell functions in TME [54]. TME includes not only tumor constituents but also some nontumor constituents such as stromal and immune cells. The proportions of stromal and immune cells and the purity of tumors can be evaluated by ESTIMATE algorithm. In this work, the estimated score of subtype3 was significantly higher than those of other three subtypes, suggesting the lowest tumor purity in subtype3. Specifically, the immune score of subtype3 was the highest, followed by that of subtype1, and subtype2 had the lowest immune score. As for the stromal score, the score of subtype3 was also the highest, that of subtype1 was the lowest, and those of subtype2 and subtype4 were moderate (Figures 3(d)–3(f)). Besides, correlation analysis between 34 prognostic OSRGs and three kinds of ESTIMATE scores was also conducted (Spearman, *P* < 0.05). According to the research results, the three scores were positively correlated with over half of the prognostic OSRGs, including *CD38*, *FZD1*, *HGF*, *IL18BP*, *JAK2*, *PDGFRA*, *SIRPA*, and *TRPM2*, while prognostic OSRGs like *PRDX6* and *SIGMAR1* had negative correlation with these scores (Figure 3(g)).

**Figure 4:**
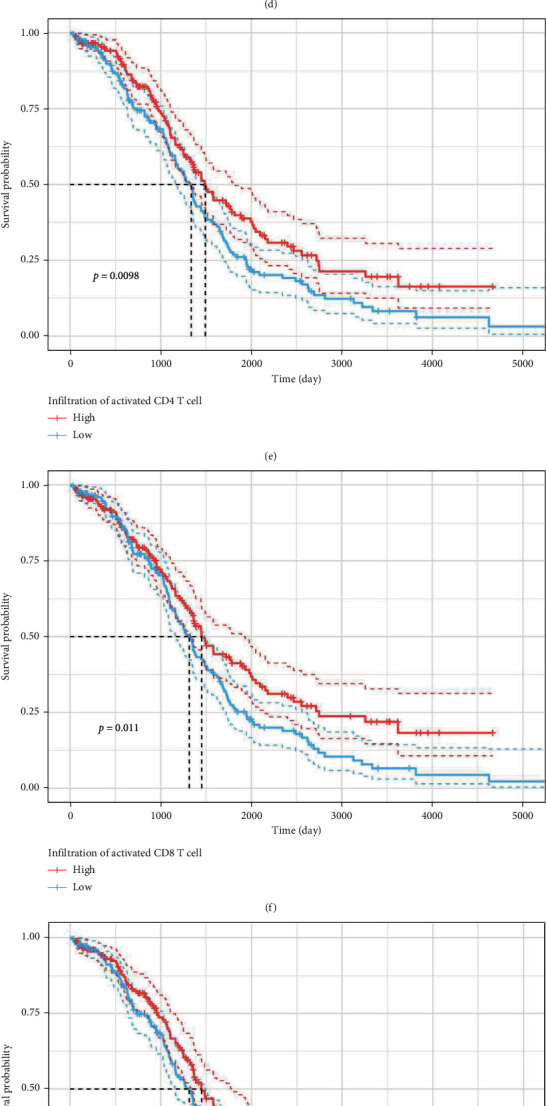
Immune cell infiltration analysis among the four OC subtypes. (a) The heatmap showing immune infiltration analysis on 28 kinds of immune cells based on the ssGSEA algorithm among the four subtypes. (b) The significant test of immune cell infiltration levels among the four subtypes by the Kruskal-Wallis test. (c) The correlation matrix between 28 immune cells and 34 prognostic OSRGs. (d–k) The Kaplan-Meier survival analysis on different infiltration levels of immune cells in OC. ^∗^: *P* ≤ 0.05; ^∗∗^: *P* ≤ 0.01; ^∗∗∗^: *P* ≤ 0.001.

Since the immune score of subtype3 was significantly higher than those of the other three subtypes, the difference in TIME among OC subtypes was subsequently analyzed by ssGSEA. In general, compared with subtype1 and subtype4, the immune cell infiltration level of subtype3 was the highest, while that of subtype2 was the lowest. Furthermore, the infiltration levels of some immune cells such as activated CD4 T cells, activated CD8 T cells, effector memory CD8 T cells, gamma delta T cells, immature B cells, MDSC, and type2 T helper cells of subtype1 were second only to those of subtype3 (Figures 4(a) and 4(b)). Thereafter, the Spearman correlation analysis was performed to examine the relations between the infiltration degrees of 28 immune cells and the expression levels of 34 prognostic OSRGs. As a result, some genes like *CD38*, *HGF*, *IL18BP*, *JAK2*, and *SIRPA* were positively correlated with almost all the 28 immune cells, while *PRDX6* was negatively correlated with these immune cells (Figure 4(c)). According to our study, one OSRG was correlated with the infiltration degrees of most immune cells in the same trend (positive or negative), but for one kind of immune cell, its correlation with the expression levels of different OSRGs did not satisfy this pattern. These findings revealed that the differences in immune cell infiltration levels of OC patients might be caused by key OSRGs. Finally, survival analysis indicated that patients with higher infiltration levels of some antitumor immune cells (such as natural kill cells, activated CD4 T cells, and activated B cells) had better prognosis than those with lower infiltration levels of these immune cells. Specifically, there were significant differences in the survival ability of patients separately divided by the infiltration levels of activated B cells, activated CD4 T cells, activated CD8 T cells, and natural kill T cells (Figures 4(d)–4(k)).

### 3.4. Establishment and Verification of the OSRG-Based Prognostic Signature

**Figure 5:**
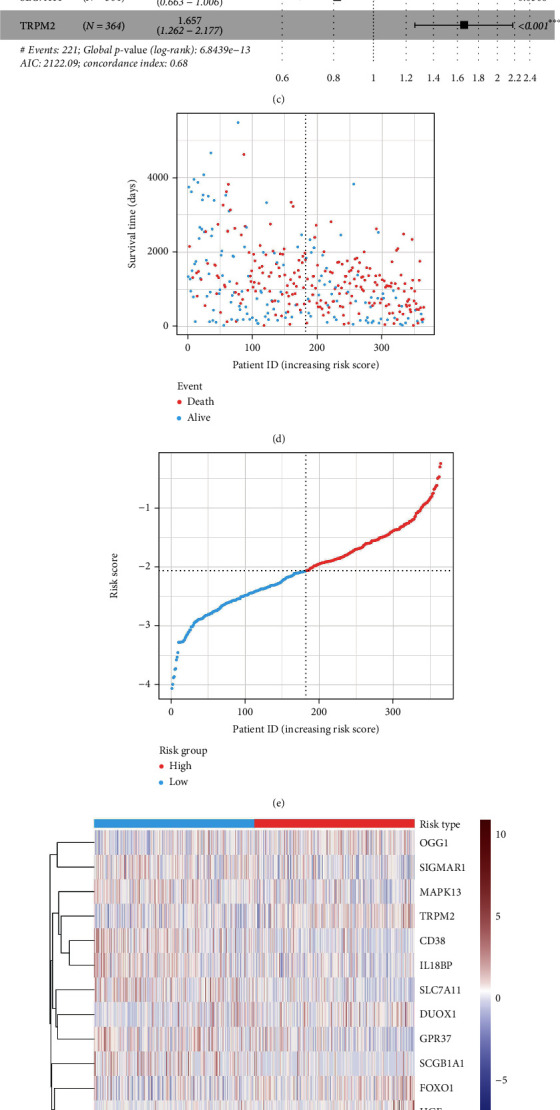
Establishment of a prognostic signature incorporating 14 OSRGs for OC patients. (a, b) LASSO algorithm for shrinking the number of OSRGs and selecting the most valuable genes from all those identified prognostic OSRGs. (c) Forest map showing more details of 14 prognostic factors selected after multivariate Cox regression analysis. (d) Scatter diagram revealing the survival-death status of all patients ranked by risk scores. (e) Risk scores of patients in two risk groups. (f) Heatmap displaying the expression levels of all the signature genes. (g) The Kaplan-Meier analysis on OC patients significantly separated into high- and low-risk groups. (h) Time-dependent ROC curves showing the OSRG-based prognostic signature of OC.

By adopting TCGA dataset as the training dataset, the LASSO regression was carried out to shrink the number of genes from those 34 identified prognostic OSRGs. On account of the coefficient of each gene, the prognostic signature incorporating OSRGs was constructed by retaining the 14 most valuable OSRGs (Figures 5(a) and 5(b)). Thereafter, the risk score of each OC patient was calculated by multivariate Cox regression analysis (Figure 5(c)) by the following equation:

(2) $$\begin{array}{c} \text{Risk score}=\left(-0.22528\right)\times \text{ARL}6\text{IP}5+\left(-0.31889\right)\times \text{CD}38+0.20716\times \text{DUOX}1+0.19167\times \text{FOXO}1+\left(-0.27031\right)\times \text{GPR}37+0.32934\times \text{HGF}+\left(-0.32978\right)\times \text{IL}18\text{BP}+\left(-0.21529\right)\times \text{MAPK}13+0.33075\times \text{OGG}1+0.40601\times \text{PLA}2\text{R}1+\left(-0.14751\right)\times \text{SCGB}1\text{A}1+\left(-0.20199\right)\times \text{SIGMAR}1+\left(-0.20294\right)\times \text{SLC}7\text{A}11+0.50517\times \text{TRPM}2. \end{array}$$

There were eight favorable prognostic genes and six poor prognostic genes incorporated in this signature, and a variety of OSRGs such as *CD38*, *FOXO1*, *HGF*, *IL18BP*, and *TRPM2* were closely involved in the previous studies regarding cancer subtypes and immune landscape.

Afterwards, depending on the median risk score, OC patients were divided into high- and low-risk groups. As observed from the risk score chart and the survival-death status chart, this signature clearly divided patients into two risk groups, and the expression levels of 14 signature genes were visualized by heatmap (Figures 5(d)–5(f)). In particular, upon KM curve analysis, low-risk patients had remarkably greater survival ability than high-risk patients (*P* < 0.0001) (Figure 5(g)). Moreover, the area under the curve (AUC) values were equal to 0.71, 0.76, and 0.85 in 3, 5, and 7 years, respectively, indicating that the OSRG-based prognostic signature exhibited good accuracy (Figure 5(h)).

**Figure 6:**
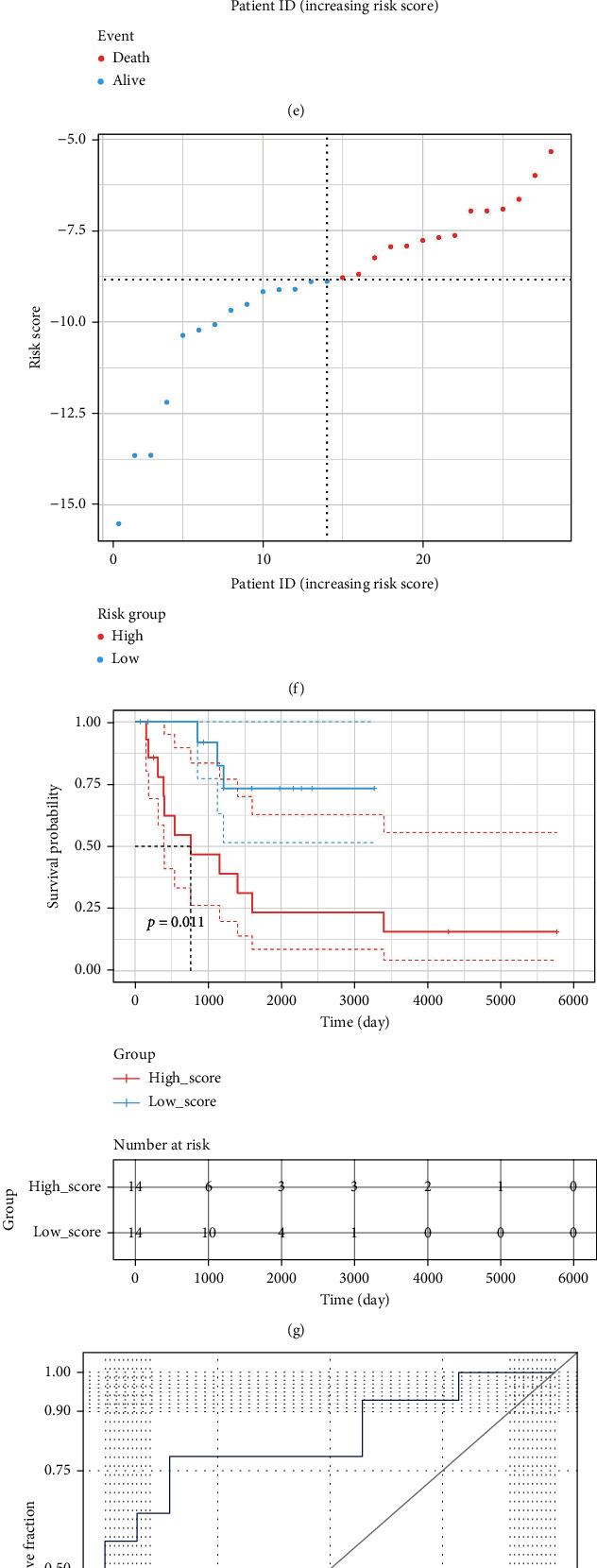
Verification of the prognostic signature in external datasets. (a–d) Verification of the as-constructed prognostic signature in GSE14764 dataset by the survival-death status diagram, Kaplan-Meier analysis, and ROC analysis. (e–h) Verification of the as-constructed prognostic signature in GSE23554 dataset by the survival-death status diagram, Kaplan-Meier analysis, and ROC analysis. (i–l) Verification of the as-constructed prognostic signature in GSE63885 dataset by the survival-death status diagram, Kaplan-Meier analysis, and ROC analysis.

To testify the stability of the as-constructed signature, GSE14764, GSE23554, and GSE63885 datasets were selected as the external datasets. In each external dataset, the OS-related risk scores clearly divided OC patients into high- and low-risk groups. To be specific, KM survival curves indicated significant difference in patient prognosis between the two risk groups (*P* < 0.05), and the AUC values were 0.79, 0.84, and 0.73 in GSE14764, GSE23554, and GSE63885 datasets, respectively (Figures 6(a)–6(l)). On the whole, the OSRG-based prognostic signature displayed good accuracy and robustness in evaluating the survival ability of OC patients.

### 3.5. Differences in Gene Set Enrichment and Somatic Mutation between the High- and Low-Risk Groups

**Figure 7:**
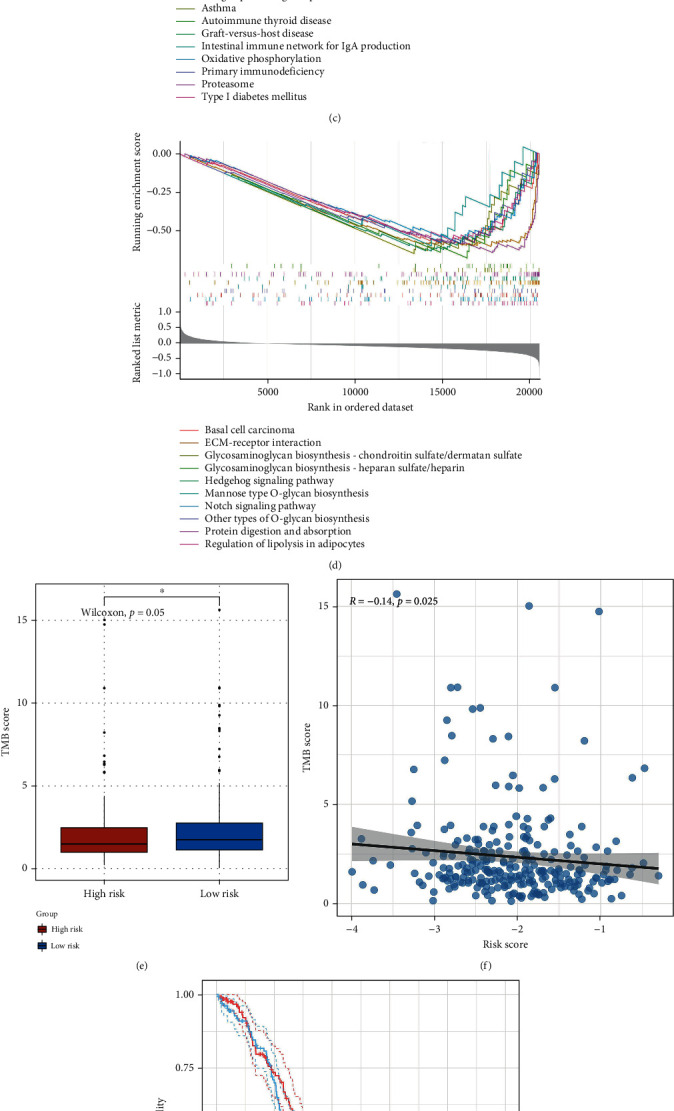
Details of gene set enrichment and somatic mutation analyses between the high- and low-risk groups. (a, b) Visualization of DEGs between the two risk groups by heatmap and volcano plot. (c) The top 10 KEGG signaling pathways enriched in the low-risk group sorted by the enrichment score. (d) The top 10 KEGG signaling pathways enriched in the high-risk group sorted by the enrichment score. (e) Significant difference in TMB level between the two risk groups. (f) Negative correlation between TMB value and OS-related risk score. (g) Prognostic value of TMB score. (h) Oncoprint showing the mutation types of the top 25 driver genes and their distributions in different risk groups. ^∗^: *P* ≤ 0.05.

To explore the biological differences between the two risk groups, GSEA was first utilized to study the biological signaling pathways involved in different groups. DEGs between the two groups were identified by R package “limma” (Figures 7(a) and 7(b)), then genes were sorted out in a descending order by log2 FC value, and GSEA was later performed with R package “clusterProfiler.” According to the research results, a total of 22 signaling pathways were enriched in the low-risk group while 97 in the high-risk group. Specifically, the enriched KEGG pathways in the low-risk group mainly included allograft rejection, viral protein interaction with cytokine and cytokine receptor, intestinal immune network for IgA production, autoimmune thyroid disease, primary immunodeficiency, antigen processing and presentation, natural killer cell mediated cytotoxicity, graft-versus-host disease, and systemic lupus erythematosus. Meanwhile, the enriched KEGG pathways in the high-risk groups mainly included glycosaminoglycan biosynthesis, protein digestion and absorption, hedgehog signaling pathway, EGFR tyrosine kinase inhibitor resistance, ECM-receptor interaction, Notch signaling pathway, TGF-beta signaling pathway, PI3K-Akt signaling pathway, mTOR signaling pathway, and Wnt signaling pathway (Supplemental Table 2 and Figures 7(c) and 7(d)). Consequently, many immune-related signaling pathways and immune-related diseases were enriched in the low-risk group, while a variety of cancers and carcinogenic pathways were enriched in the high-risk group. In addition, the signaling pathways based on each signature gene were investigated in order to know if these genes can be considered as potential biomarkers for clinical application. The results revealed that there were various significant signaling pathways related to each signature gene, and the shared signaling pathways mainly included allograft rejection, antigen processing and presentation, apoptosis, cell adhesion molecules, B cell receptor signaling pathway, chemokine signaling pathway, and PD-L1 expression and PD-1 checkpoint pathway in cancer (Supplemental Table 3-16).

TMB is increasingly suggested to have important clinical significance. The higher nonsynonymous mutation burden is linked to the prolonged progressive free survival [55], and patients with higher TMB level have better response to immunotherapy [56]. Therefore, the internal relation between TMB and OS-related risk score was further analyzed in the present work. First of all, the TMB level of OC patients from TCGA somatic mutation data was assessed by R package “maftools.” As shown in Figures 7(e) and 7(f), TMB level of patients in the high-risk group was remarkably lower than that in the low-risk group (Wilcoxon, *P* = 0.05). Besides, correlation analysis demonstrated the negative correlation between TMB and OS-related risk score (Spearman, *P* < 0.05). The KM survival analysis verified that the higher TMB level predicted better prognosis to a certain extent, although it did not reach statistical significance (Figure 7(g)). Thereafter, somatic mutation between the two groups was further assessed in details. It was more likely that patients underwent single nucleotide polymorphism (SNP) that transferred from cytosine to thymine, but the frequency of each mutation and genes with higher mutation frequency were significantly different between the high-and low-risk groups (Supplemental Figure 2a-b). Additionally, the driver genes in OC were evaluated, and later mutation types of the top 25 driver genes and their distributions in OC patients were visualized by Oncoprint (Supplemental Figure 2c and Figure 7(h)). Upon Fisher's exact test, differences in mutation frequencies of *HMCN1*, *TRANK1*, *KAT6B*, *JAG1*, and *TCOF1* were significant between the two groups (*P* < 0.05) (Supplemental Table 17). In conclusion, these results provide novel insights for further study on OS and gene mutations in OC.

### 3.6. Prediction of Immunotherapy Response between the High- and Low-Risk Groups

**Figure 8:**
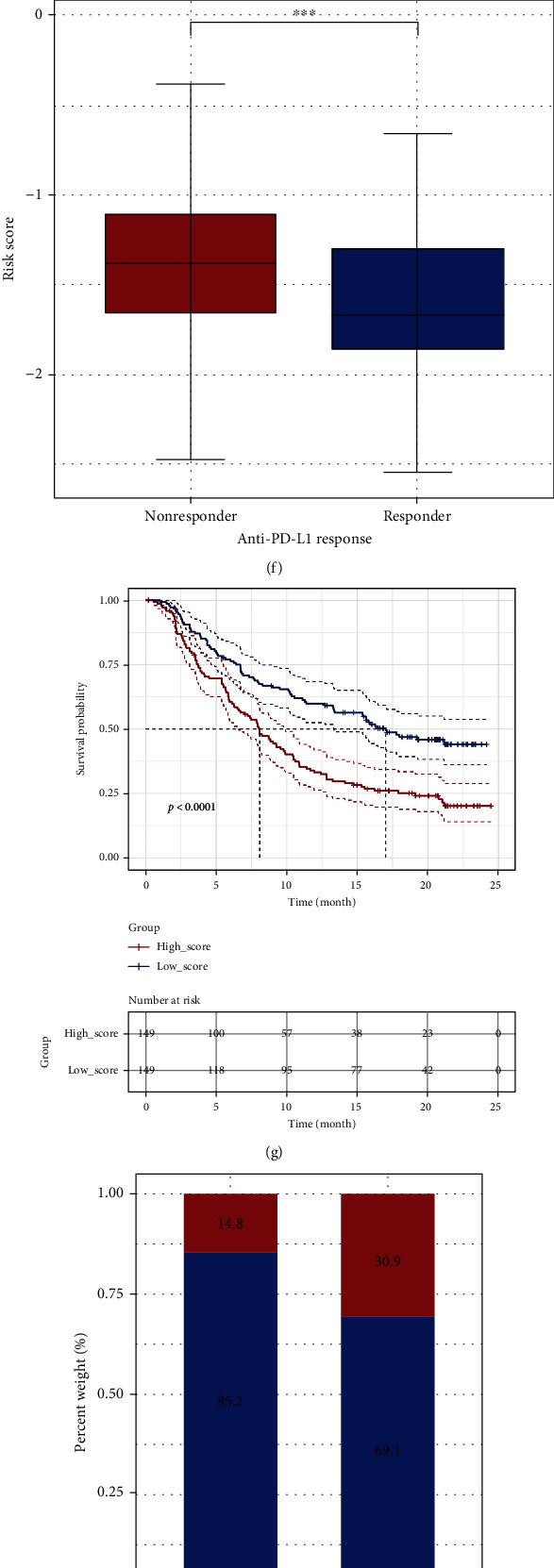
The prognostic value of OSRG signature in immunotherapy. (a) The heatmap showing the expression levels of 16 immune checkpoint genes (ICGs). (b) The significant test regarding the expression levels of 16 ICGs between the two risk groups by the Wilcoxon test. (c) The Spearman correlation analysis between the OS-related risk score and ICGs. (d) Difference in the TIDE score between the high- and low-risk groups. (e) The positive correlation between the OS-related risk score and TIDE score. (f–i) In IMvigor210 cohort, patients receiving anti-PD-L1 immunotherapy had different response rates between the high- and low-risk groups, and their immune phenotypes were also significantly different in term of the OS-related risk score. (j–m) In GSE78220 cohort, patients receiving anti-PD-1 immunotherapy also had different response rates between the high- and low-risk groups. ns: not significant; ^∗^: *P* ≤ 0.05; ^∗∗^: *P* ≤ 0.01; ^∗∗∗^: *P* ≤ 0.001.

As suggested in the previous studies, patients with higher TMB status have better clinical responses to anti-PD-1/L1 immunotherapy [56, 57]. Moreover, OS can not only affect the expression of ICGs in cancer cells but also act as a key mediator for ICI resistance [58, 59]. In our study, differences in signaling pathways and TMB level between patients in the high- and low-risk groups revealed the potential association between OS-related risk score and immunotherapy, which inspired us to explore further studies on immunotherapy. First of all, the study on key ICGs unveiled that, apart from PD-1, the expression levels of most ICGs (including *CD27*, *CD58*, *CTLA4*, *ICOS*, *IDO1*, *LAG3*, *PD-L1*, *PD-L2*, and *TIGIT*) in the high-risk group were remarkably lower than those in the low-risk group (Wilcoxon, *P* < 0.05) (Figures 8(a) and 8(b)). The corresponding results are reflected in Figure 8(c), showing that the expression of most ICGs (except for *PD-1* and *B7-H3*) was negatively correlated with OS-related risk score. In addition, relations between the expression levels of ICGs and prognosis were also analyzed; as a result, the higher expression levels of *CD27*, *IDO1*, *PD-L2*, *TIGIT*, *ICOS*, and *LAG3* predicted the relatively favorable prognosis (Supplemental Figure 3a-p). In particular, there was significant difference in the effects of these 6 ICGs on prognosis between the two risk groups (Supplemental Figure 4a-f), demonstrating that the impacts of some ICGs on prognosis might be affected by the OSRGs model. Furthermore, according to the previous studies, the higher expression of some ICGs may act as one of biomarkers for the enhanced ICI sensitivity [60, 61]. Therefore, the TIDE algorithm was adopted in the present work to predict the ICI responses in patients. As discovered, the TIDE score of patients in the high-risk group was significantly higher than that in the low-risk group (Figure 8(d)), and the TIDE score was positively correlated with the OS-related risk score (Figure 8(e)). Collectively, it is more possible for patients in the high-risk group to undergo immune escape, while those in the low-risk group are more likely to benefit from immunotherapy.

Based on the above analyses, this work hoped to further evaluate the clinical immunotherapeutic response rates between patients in the high- and low-risk groups. Due to the lack of public clinical information on anti-PD-1/L1 immunotherapy in OC, another two published datasets, namely, IMvigor210 and GSE78220, which recorded gene expression profiles and clinical information of patients in metastatic urothelial cancer and metastatic melanoma after anti-PD-L1 checkpoint inhibition therapy and anti-PD-1 checkpoint inhibition therapy, respectively [62, 63], were selected. Patients receiving anti-PD-L1 checkpoint inhibition therapy in IMvigor210 cohort were divided into high- and low-risk groups according to the OSRG-based prognostic signature. The results revealed that patients responding to immunotherapy had a remarkably lower OS-related risk score than the nonresponders (Figure 8(f)) and that patients in the low-risk group had better prognosis (Figure 8(g)). More importantly, there were remarkably more patients responding to immunotherapy in the low-risk group than in the high-risk group, accounting for 30.9% and 14.8%, respectively (Figure 8(h)). Moreover, upon the Kruskal-Wallis test, there was significant difference in the OS-related risk score of patients with three different immune phenotypes (desert, excluded, and inflamed) (Figure 8(i)). Similarly, in GSE78220 cohort, the OS-related risk score of patients responding to immunotherapy was significantly lower than that of the nonresponders, and there were markedly more patients in the low-risk group than in the high-risk group, occupying 69.2% and 35.7%, separately, although there was no significant correlation between disease status and OS-related risk score (Figures 8(j)–8(m)). In general, these results illustrated the latent value of the OSRG-based prognostic signature in immunotherapy and demonstrated the role of OSRG-based prognostic signature as a potent predictor for immunotherapeutic response to a certain extent.

### 3.7. The Role of OSRG Model in Chemotherapy

**Figure 9:**
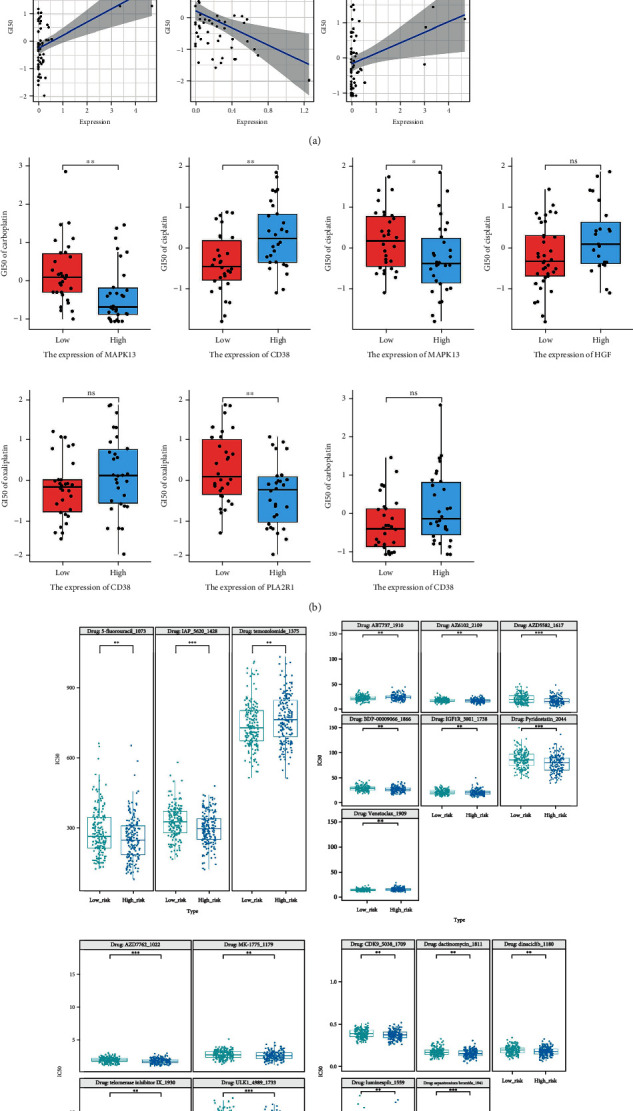
The prognostic value of OSRG signature in chemotherapy. (a) The Spearman correlation analysis between expression levels of signature genes and GI_50_ values of drugs in the CellMiner database. (b) The boxplots showing the difference in platinum sensitivity between the two groups divided by the expression levels of corresponding signature genes. (c) Pan-drug sensitivity analysis between the patients in the high- and low-risk groups based on the GDSC database. ns: not significant; ^∗^: *P* ≤ 0.05; ^∗∗^: *P* ≤ 0.01; ^∗∗∗^: *P* ≤ 0.001.

Compared with immunotherapy, platinum-based cytotoxic chemotherapy is applied in the treatment of most advanced OC patients, but platinum-based drug resistance and the associated numerous adverse reactions are the common causes of death in patients [2, 64]. Therefore, this work is aimed at investigating the role of OSRGs model in chemotherapy and providing some novel strategies for OC patients. Three platinum-based drugs (cisplatin, carboplatin, and oxaliplatin) were selected based on the CellMiner database, and the relations between GI_50_ values of these drugs and the expression levels of 14 prognostic signature genes were analyzed. As a result, the expression levels of *MAPK13*, *CD38*, *HGF*, and *PLA2R1* were correlated with the GI_50_ values of the three platinum-based drugs (Spearman, *P* < 0.05) (Figure 9(a)). Besides, boxplots were drawn to show the differences in GI_50_ values of the three platinum-based drugs at different expression levels of signature genes (Figure 9(b)). Moreover, the corresponding data of OC cell lines in GDSC (version 2) were selected as training datasets, and the IC_50_ values of 181 drugs in patients were predicted by R package “oncoPredict.” According to the research results, there were 19 drugs with significant differences in IC_50_ values between the two groups (Wilcoxon, *P* < 0.01), and the IC_50_ values of most of these drugs of the low-risk group were noticeably higher than those of the high-risk group, suggesting that patients in the high-risk group were more likely to benefit from some specific agents (Figure 9(c)).

## 4. Discussion

OC is a common and fatal female malignancy, and numerous studies have been conducted to explore its molecular mechanisms and therapeutic strategies. An increasing number of studies have indicated that OS is an essential factor that affects the occurrence and progression of OC. Compared with normal samples, OS level significantly increases in OC, which also shows the protumor and prometastasis effects [65]. Besides, OS has been extensively verified to affect the chemoresistance and immunotherapy in cancer patients [66, 67]. Consequently, it is necessary to investigate the effect of OSRGs on the prognosis of OC patients. With the advancement of high-throughput technologies and the development of multiomics databases, an increasing number of studies have utilized bioinformatics methods to establish prognostic models based on gene expression profiles, which can better predict the survival of cancer patients and identify more powerful therapeutic targets [68]. However, to our knowledge, no existing study has been conducted to predict the prognosis of OC patients by constructing the OSRG-based gene signature. Therefore, to fully understand the role of OS in OC, 423 OSRGs in OC were analyzed in this study. After identifying 34 prognostic OSRGs, TCGA and GEO databases were chosen to establish and verify the OSRG-based prognostic signature. Moreover, the biological functions of these prognostic OSRGs and their effects on OC subtypes were also examined. Afterwards, based on the prognosis of OC patients at the individual level assessed by the OS-related risk score, the signaling pathways, TMB, and ICGs between different risk groups were systemically analyzed, which laid the foundation for researchers to further understand the role of OS in tumors and develop more effective immunotherapy and chemotherapy treatments for OC patients.

After univariate Cox regression analysis, LASSO analysis, and multivariate Cox regression analysis on 423 OSRGs, the prognostic signature containing 14 genes (*ARL6IP5*, *CD38*, *DUOX1*, *FOXO1*, *GPR37*, *HGF*, *IL18BP*, *MAPK13*, *OGG1*, *PLA2R1*, *SCGB1A1*, *SIGMAR1*, *SLC7A11*, and *TRPM2*) was constructed. Later, this as-constructed signature was comprehensively analyzed. As revealed by the KM survival analysis, the OS-related risk score was noticeably associated with the overall survival rate of OC patients, and the AUC values of t-ROC curves were all higher than 0.7 after three years, indicating the high accuracy of this signature. Moreover, three independent external GEO datasets also showed that this signature effectively predicted the clinical outcomes of OC patients. More importantly, numerous previous studies have confirmed that these signature genes play crucial roles in multiple cancers, including OC. For instance, the ADP ribosylation factors like GTPase 6 interacting protein 5 (*ARL6IP5*), a kind of microtubule-associated protein, can reduce the resistance to cisplatin in OC cells by suppressing DNA repair protein and promoting apoptosis. In addition, *ARL6IP5* also acts as a significant prognostic factor and tumor suppressor in OC [69]. Forkhead box protein O1 (*FOXO1*) is the downstream target of PI3K/Akt signaling pathway, which is upregulated in EOC tissues, and its overexpression predicts the poor prognosis. Besides, the proliferation and migration abilities will significantly change in EOC cells after *FOXO1* knockdown [70]. *FOXO1* has been confirmed to be significantly upregulated in paclitaxel-resistant cells and tissues from chemoresistant OC patients [71]. Hepatocyte growth factor (*HGF*), a significant component of HGF/cMET pathway, is highly expressed in ascites and serum of OC patients. Some studies have suggested that the increased *HGF* level in serum usually predicts the shorter overall survival, which lays a theoretical foundation for the study on drugs targeting HGF/cMET pathway [72, 73]. 8-Oxoguanine DNA glycosylase (*OGG1*) is one of the most important proteins encoded in the base excision repair of DNA, which plays a significant role in correcting the OS-induced DNA damage. SNP in *OGG1* has been previously suggested to contribute to more serious nuclear DNA damage, and the rs2304277 variant in *OGG1* can increase the risk of OC in *BRCA1* mutation carriers [74, 75]. Solute carrier family 7 member 11 (*SLC7A11*) is a potential target for the chemosensitivity to various drugs in cancer [76]. The expression of *SLC7A11* is remarkably downregulated in paclitaxel-resistant OC cells, which is associated with poor prognosis and may result from the interaction between competing endogenous RNA (ceRNA) and cell autophagy-related genes [77]. Therefore, the above results confirm that genes incorporated in this signature are not only the most characteristic biomarkers for OC but also can provide multiple latent biological targets to further explore the mechanisms of OC and explore its therapeutic strategies.

Based on the prognostic signature of OSRGs, TCGA-derived OC patients were divided into high- and low-risk groups. Next, this work explored the biological differences between the two risk groups, which provided certain insights into the treatment of OC. GSEA results showed that some immune-related signaling pathways such as allograft rejection, viral protein interaction with cytokine and cytokine receptor, antigen processing and presentation, and intestinal immune network for IgA production were enriched in the low-risk group. Meanwhile, some pathways closely related to cancers such as Notch signaling pathway, TGF-beta signaling pathway, PI3K-Akt signaling pathway, mTOR signaling pathway, and Wnt signaling pathway were enriched in the high-risk group. In addition, TMB was negatively correlated with the OS-related risk score. Upon the Wilcoxon test, patients in the low-risk group had higher TMB levels, which generally meant that low-risk patients had better responses to immunotherapy [56].

Up to now, there are limited treatment strategies for OC. Although various chemotherapeutic agents and platinum-based adjuvant therapies have improved the prognosis of patients to some degree, the high recurrence and chemoresistance rates are still the challenges encountered by OC treatment [2]. In recent years, immunotherapy has drawn more and more attention from the researchers and become a new treatment strategy for OC [78]. Previous study has suggested that OS is related to PD-1 blockade immunotherapy [67]. Immune checkpoint blockade (ICB) is one of the most influential immunotherapies; however, only a few patients can benefit from this therapy [79]. Besides, immune microenvironment characterized by T cell clonality is reported to play important roles in clinical outcome, and one study has suggested that the expression levels of immune-related genes are related to the clonality of infiltrated T cells by T cell receptor *β* sequencing in endometrial cancer [80]. Based on the potential associations of signaling pathways and TMB level with immunotherapy in the above study, this study subsequently explored the immunotherapeutic responses of patients between different risk groups. First, it was found that most ICGs (except for *PD-1*) were upregulated in the low-risk group, which indicated that these patients were more likely to be sensitive to ICIs. When predicting the immunotherapeutic response, immune dysfunction and exclusion for each patient were estimated through TIDE module. As a result, patients in the high-risk group were more susceptible to immune dysfunction and escape, while those in the low-risk group were more likely to benefit from immunotherapy. Finally, by adopting IMvigor210 and GSE78220 datasets, it was verified that the OS-related risk score was able to predict the clinical response to anti-PD-1/L1 immunotherapy. Such result indicated that the OS-related risk score of responders in both datasets was remarkably lower than that of nonresponders, and patients in the high-risk group had remarkably lower responses than the low-risk group, which revealed that patients with lower OS-related risk score might be more likely to benefit from anti-PD-1/L1 immunotherapy.

Additionally, this study analyzed the value of OSRG signature in OC chemotherapy. OC has been widely reported to benefit from platinum-based drugs, and one previous study has found that the levels of some biomarkers of OS for instance 8-isoprostane were significantly increased in ascites fluid of EOC patients among the platinum-sensitive group, platinum-resistant group, and platinum-refractory group [81]. Firstly, the relations between signature genes and GI_50_ values of platinum-based drugs at the gene expression level in the CellMiner database were analyzed. For example, mitogen-activated protein kinase 13 (*MAPK13*) is an important part of MAP kinase signaling pathway, which is previously reported to be overexpressed in gynecological cancer stem cells including ovarian cancer [82] compared with adjacent normal tissues. Further, it can be used as a signature gene to predict the chemotherapeutic response of cisplatin [83]. In our study, the expression level of *MAPK13* was negatively correlated with the GI_50_ values of carboplatin and cisplatin, suggesting that the therapeutic effects of carboplatin and cisplatin might be improved with the increase in *MAPK13* expression level. On the other hand, cluster of differentiation 38 (*CD38*) is an emerging therapeutic target [84]; when *CD38* expression level is upregulated, cisplatin, carboplatin, and oxaliplatin all show an upward trend in their GI_50_ values, which may lead to platinum resistance. Afterwards, this study investigated differences in sensitivity to chemotherapeutic agents of patients at the individual level, which revealed that the sensitivities of some drugs such as 5-fluorouracil, temozolomide, venetoclax, telomerase inhibitor, luminespib, and dactinomycin were different between the high- and low-risk groups. 5-Fluorouracil (5-FU), an extensively used antitumor drug, can enhance the cytotoxicity induced by cisplatin and radiotherapy through suppressing DNA repair [85], but its overall response rate to cancers is low when used alone [86]; in this regard, 5-FU is often used in combination with other antitumor drugs. For instance, study has confirmed that a three-drug combination strategy (OBP-801/YM753, 5-FU, and paclitaxel) better inhibits OC cell growth than each single-agent or the two-agent combination strategies [87]. Another study has reported that the combination of intraperitoneal 5-FU and cisplatin is a potent strategy for the treatment of relapsed or residual EOC [88]. Venetoclax is an effective agent for chronic lymphocytic leukemia (CLL) and acute myeloid leukemia (AML) [89, 90], and its minimal combination with paclitaxel has a great effect on OC cells [91]. In conclusion, these analyses, combined with previous immunotherapy strategies, can shed new lights on the more accurate individualized treatment for OC patients.

Furthermore, since the disease stage of OC is one of the most important clinical characteristics, cancer subtypes were also analyzed based on OSRGs in this study. According to the results, TME and immune cell infiltration levels were different among these four OC subtypes, and the expression levels of some key prognostic OSRGs were remarkably linked to the infiltration levels of some immune cells, demonstrating that OS might be concerned with the regulation of TME. Interestingly, this analysis revealed that patients with higher infiltration levels of immune cells had superior prognosis, which seemed to be contradictory with the previous survival analysis of different subtypes suggesting that patients of subtype3 showed poor prognosis while those of subtype2 had better prognosis. Such observation further proved the complexity of TME, which indicated that antitumor immune cells were not the best predictor of patient prognosis and that patient prognosis might also be affected by the combination of protumor immune cells, stromal components, and non-TME factors.

Generally, this study explores the prognosis of OC patients based on OSRGs, which is beneficial to guide the individual treatment for OC patients. However, some limitations should be noted in this study. First, due to lack of more public resources and the impact of batch effects, the number of OC patients in our study was limited, so larger datasets are necessary to validate our prognostic signature in the future. Besides, due to the lack of normal samples in TCGA, data from OC samples and normal samples were not compared. Furthermore, due to the scarcity of clinical information on immunotherapy in OC, it was impossible to accurately evaluate the effect of immunotherapy of the OSRG-based prognostic signature in OC. Finally, the results of this study should be further testified by biological experiments and clinical trials.

## 5. Conclusions

In this study, a novel and reliable prognostic signature integrating 14 OSRGs is constructed, and the accuracy of this signature is well validated in several external databases. After comprehensively investigating the association of OS-related risk score with multiple biological processes, these OSRGs are confirmed to act as potential biomarkers of OC. Besides, associations between the OS-related risk score and the impact of immunotherapy are investigated thoroughly by combining immune checkpoint analysis, TIDE algorithm, and various databases containing immunotherapeutic response information, which reveals that OC patients with lower OS-related risk score are more likely to benefit from anti-PD-1/L1 immunotherapy. In addition, the sensitivity of chemotherapy is different in OC patients with low- and high-risk stage. Generally, this study is beneficial to assist researchers to understand the underlying pathogenesis of OC and shed new lights on the clinical treatment of OC.

## Data Availability

The data from TCGA and GEO datasets used to support the findings of this study are available from the corresponding author upon request.

## Supplementary Materials

Supplementary 1 Supplemental Figure 1: complementary figures of cancer subtype analysis. (a–h) Consensus heatmap showing various subtype numbers (*k* = 2 − 10, *k* = 4 is shown in Figure 3). (i) The legend of consensus matrix, where the color of consensus heatmap from white to blue represents the value of matrix from 0 to 1. (j–k) The cumulative distribution function (CDF) curves and their delta area of different subtypes. (l) The tracking plot of different subtypes. Supplemental Figure 2: complementary figures of tumor mutation burden analysis. (a) Detailed information of somatic mutation of high-risk patients. (b) Detailed information of somatic mutation of low-risk patients. (c) Oncoprint showing the mutation types of the top 25 driver genes and their distributions in all TCGA-derived OC patients. Supplemental Figure 3: assessment of the prognostic significance of 16 immune checkpoint genes (ICGs). (a–p) Prognostic significance of each ICG, and 6 ICGs were selected as prognostic factors. The upregulated expression of these 6 ICGs predicted the favorable prognosis. Supplemental Figure 4: further assessment of the prognostic significance of 6 prognostic immune checkpoint genes (ICGs). (a–f) The Kaplan-Meier survival analysis indicating the noticeably disparate impacts of these 6 ICGs (*CD27*, *IDO1*, *PD-L2*, *TIGIT*, *ICOS*, and *LAG3*) on prognosis between the high- and low-risk groups.

Supplementary 2 Supplemental Table 1: detailed information of 34 prognostic OSRGs after univariate Cox regression analysis.

Supplementary 3 Supplemental Table 2: detailed results of gene set enrichment analysis (GSEA).

Supplementary 4 Supplement Table 3: signaling pathway analysis in regard to ARL6IP5.

Supplementary 5 Supplement Table 4: signaling pathway analysis in regard to CD38.

Supplementary 6 Supplement Table 5: signaling pathway analysis in regard to DUOX1.

Supplementary 7 Supplement Table 6: signaling pathway analysis in regard to FOXO1.

Supplementary 8 Supplement Table 7: signaling pathway analysis in regard to GPR37.

Supplementary 9 Supplement Table 8: signaling pathway analysis in regard to HGF.

Supplementary 10 Supplement Table 9: signaling pathway analysis in regard to IL18BP.

Supplementary 11 Supplement Table 10: signaling pathway analysis in regard to MAPK13.

Supplementary 12 Supplement Table 11: signaling pathway analysis in regard to OGG1.

Supplementary 13 Supplement Table 12: signaling pathway analysis in regard to PLA2R1.

Supplementary 14 Supplement Table 13: signaling pathway analysis in regard to SCGB1A1.

Supplementary 15 Supplement Table 14: signaling pathway analysis in regard to SIGMAR1.

Supplementary 16 Supplement Table 15: signaling pathway analysis in regard to SLC7A11.

Supplementary 17 Supplement Table 16: signaling pathway analysis in regard to TRPM2.

Supplementary 18 Supplemental Table 17: difference in mutation frequency of driven genes between the high- and low-risk patients by Fisher's exact test.
