# Supplementary material for: Identifying the Role of Oxidative Stress-Related Genes as Prognostic Biomarkers and Predicting the Response of Immunotherapy and Chemotherapy in Ovarian Cancer
Source: Oxid Med Cell Longev. 2022 Dec 12;2022:6575534. doi: 10.1155/2022/6575534 (PMC9764017; doi:10.1155/2022/6575534)
Supplement: Supplementary 1 — Supplemental Figure 1: complementary figures of cancer subtype analysis. (a–h) Consensus heatmap showing various subtype numbers (k = 2 − 10, k = 4 is shown in Figure 3). (i) The legend of consensus matrix, where the color of consensus heatmap from white to blue represents the value of matrix from 0 to 1. (j–k) The cumulative distribution function (CDF) curves and their delta area of different subtypes. (l) The tracking plot of different subtypes. Supplemental Figure 2: complementary figures of tumor mutation burden analysis. (a) Detailed information of somatic mutation of high-risk patients. (b) Detailed information of somatic mutation of low-risk patients. (c) Oncoprint showing the mutation types of the top 25 driver genes and their distributions in all TCGA-derived OC patients. Supplemental Figure 3: assessment of the prognostic significance of 16 immune checkpoint genes (ICGs). (a–p) Prognostic significance of each ICG, and 6 ICGs were selected as prognostic factors. The upregulated expression of these 6 ICGs predicted the favorable prognosis. Supplemental Figure 4: further assessment of the prognostic significance of 6 prognostic immune checkpoint genes (ICGs). (a–f) The Kaplan-Meier survival analysis indicating the noticeably disparate impacts of these 6 ICGs (CD27, IDO1, PD-L2, TIGIT, ICOS, and LAG3) on prognosis between the high- and low-risk groups. [file 6575534.f1.docx]

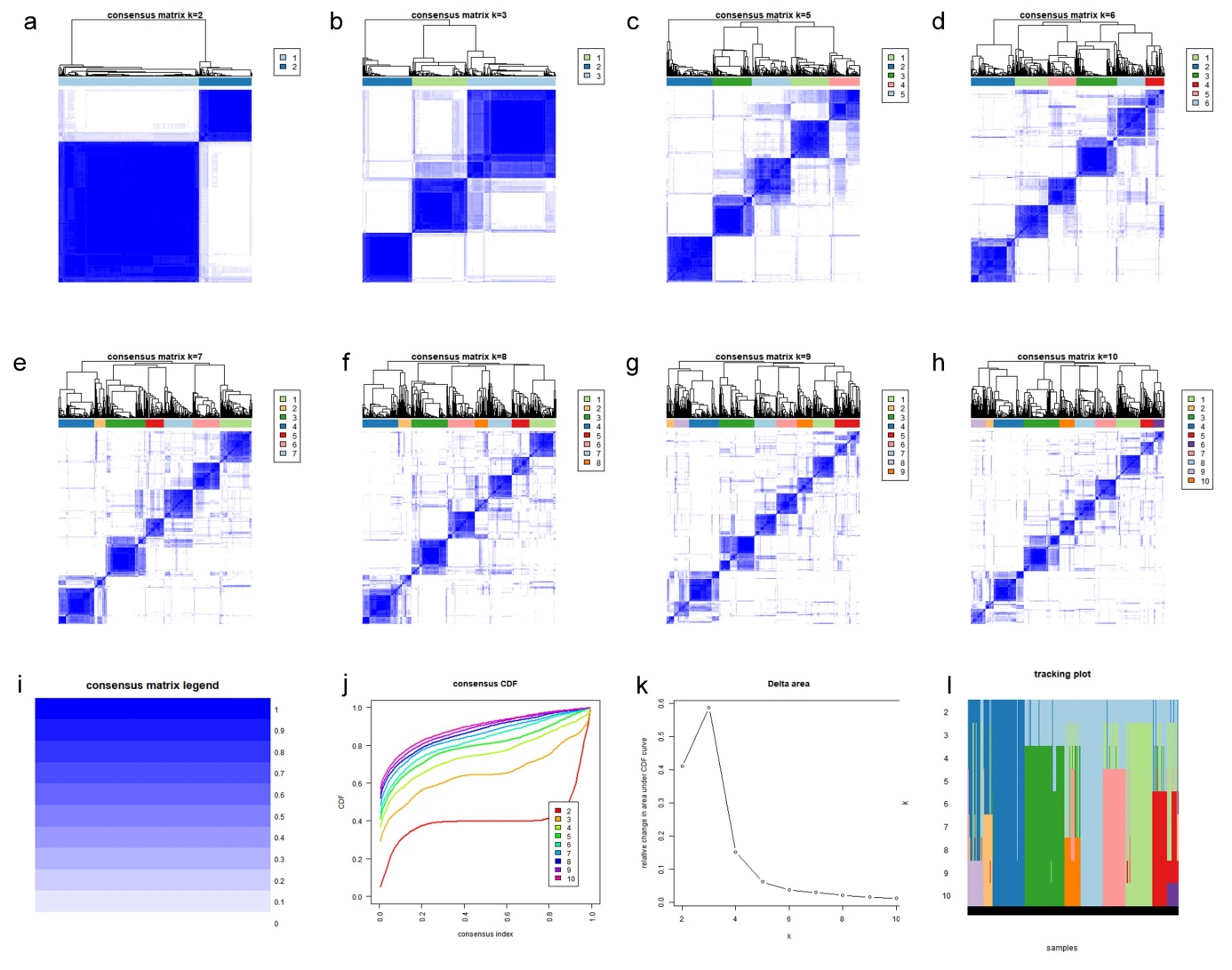


Supplemental Figure 1: Complementary figures of cancer subtype analysis. (a-h) Consensus heatmap showing various subtype numbers (k=2-10, k=4 is shown in Figure 3). (i) The legend of consensus matrix, where the color of consensus heatmap from white to blue represents the value of matrix from 0 to 1. (j-k) The cumulative distribution function (CDF) curves and their delta area of different subtypes. (l) The tracking plot of different subtypes.


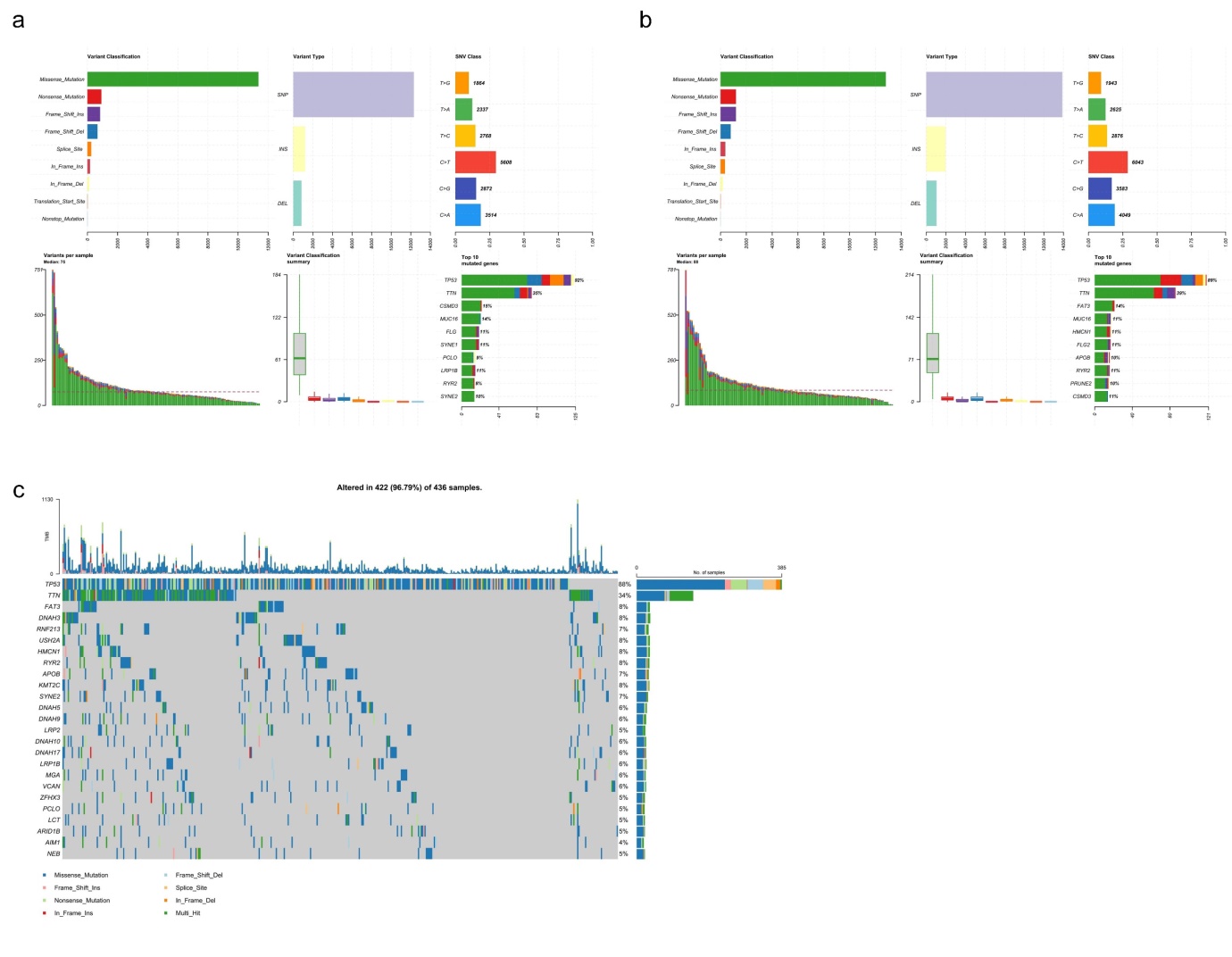


Supplemental Figure 2: Complementary figures of tumor mutation burden analysis. (a) Detailed information of somatic mutation of high-risk patients. (b) Detailed information of somatic mutation of low-risk patients. (c) Oncoprint showing the mutation types of the top 25 driver genes and their distributions in all TCGA-derived OC patients.





Supplemental Figure 3: The evaluation of prognosis for 16 immune checkpoint genes (ICGs). (a-p) The prognostic value of each ICG, and there were 6 ICGs selected as prognostic predictors. The upregulated expression of these 6 ICGs all represented favorable prognosis.





Supplemental Figure 4: Further assessment of the prognostic significance of 6 prognostic immune checkpoint genes (ICGs). (a-f) Kaplan-Meier survival analysis indicating the noticeably disparate impacts of these 6 ICGs (*CD27*, *IDO1*, *PD-L2*, *TIGIT*, *ICOS* and *LAG3*) on prognosis between high- and low-risk groups.
